# Phospholipase gamma2: A candidate genetic risk factor for Alzheimer’s disease that can alter synaptic function in DG‐perforant pathway of hippocampus

**DOI:** 10.1002/alz.084662

**Published:** 2025-01-03

**Authors:** Avishek Roy, Anael Erhardt, Severine Deforges, Christophe Mulle

**Affiliations:** ^1^ Interdisciplinary Institute for Neuroscience (UMR 5297), University of Bordeaux, Bordeaux, Gironde France

## Abstract

**Background:**

PhospholipaseC γ2 (PLCG2) is known to have direct link with genetic risk factors for Alzheimer’s like dementia (AD). PLCG2 has been previously demonstrated to have association with Aß uptake through microglia. And mostly expressed in dentate gyrus (DG) network of hippocampus.

**Method:**

WT mouse were transfected with sh‐RNA of LV‐PLCG2 (U6 promotor, GFP as reporter; shRNA) or scrambled (used as control; shNT). Whole‐cell patch‐clamp recordings were performed on acute slices with glass pipette (<3‐4MΩ) at ‐70mv, which was accompanied with morphometry of perforant pathway (PP)‐DG circuitry. Two‐tailed Mann‐Whitney test was used for statistics unless otherwise mentioned, p<0.05 was considered to be significant.

**Result:**

We found significant increase in AP_half width_ (p = 0.0274), rheobase (p = 0.0046) in shRNA on the other hand, input resistance (p = 0.0159), number of spike vs current injection (p = 0.0268) was significantly decreased. However, there were no significant change was observed in AP_onset_ (p = 0.6105), AP_amplitude_ (p = 0.7345), RMP (p = 0.7591) and firing threshold (p = 0.5247) in shRNA group when compared to shNT. mEPSC amplitude was found to be reduced (p = 0.0288) in shRNA condition, even the cumulative probability of mEPSC amplitude was also found to be significantly altered after silencing of PLCG2 gene in DG cells (p = 0.0014; Kolmogorof‐Smirnov test). mEPSC frequency of the miniature activity of transfected DG cells were did not show significant change (p = 0.7707). Sholl analysis of the DG cells expressing shRNA showed a decrease in the number of intersections vs distance from soma (P<0.05), accompanied by significant decrease in dendritic length (p = 0.0501), dendritic volume (p<0.0001) and dendritic surface area (p = 0.0273). When spine density was measured we have found a significant decrease in cumulative distribution of spine density (p<0.0001; Kolmogorof‐Smirnov test), which is tightly associated with decrease in spine length (p = 0.0209) and spine head diameter (P<0.0001). Furthermore, these dendritic and spine morphometric alteration is also accompanied with the decrease in the volume of mossy fiber gigantic bouton (mfB) with alteration in the number of filopodia per mfBs (p<0.05).

**Conclusion:**

Results from the study, reflect critical insight of PLCG2 regulating neuronal and/or synaptic activity, which may underlie the cognitive dysfunction (viz. recognition memory) and synaptic alteration to the PP‐DG circuitry seen in AD.

